# Complete sequence of mitochondrial DNA of *Gracilaria tenuistipitata* var. *liui* (Rhodophyta)

**DOI:** 10.1080/23802359.2018.1524277

**Published:** 2018-10-29

**Authors:** Xianming Tang, Yue Li, Wei Zhou Chen, Tao Liu, Jing Zhang

**Affiliations:** a Hainan Academy of Ocean and Fisheries Sciences, Haikou, People’s Republic of China;; b WuXi NextCODE, Shanghai, People’s Republic of China;; c Marine Biology Institute, Shantou University, Shantou, People’s Republic of China;; d College of Marine Life Sciences, Ocean University of China, Qingdao, People’s Republic of China;; e Qilu University of Technology, Shandong Academy of Sciences, Jinan, People’s Republic of China

**Keywords:** *Gracilaria tenuistipitata* var. *liui*, mitogenome, phylogenetic analysis

## Abstract

Here, the complete *Gracilaria tenuistipitata* var. *liui* mitogenome was determined and analyzed. The complete *G. tenuistipitata* var. *liui* mitogenome length was 25,879 bp and contained 50 genes including 24 protein-coding, 2 rRNA, and 23 tRNA genes and one unidentified open reading frame (ORF). Of the 24 protein-coding genes, 23 (95.83%) terminated with the stop codon TAA, and one (4.17%) with TAG (*rps*3 gene). All protein-coding genes in *G. tenuistipitata* var. *liui* started with ATG codon. Mitogenome phylogenetic analysis revealed that *G. tenuistipitata* var. *liui* firstly clustered together with *Gracilaria tenuistipitata.* The complete mitogenome sequence would help in understanding *Gracilaria* evolution.

*Gracilaria tenuistipitata* var. *liui* Zhang and Xia (Gracilariaceae, Rhodophyta) is found locally in South-East Asia and China (Phang 2006) and extensively cultivated in southern China (Chiang 1980). Being the sources for the phycocolloids and food, the marine red alga *Gracilaria tenuistipitata* var. *liui* Zhang and Xia is economically important (Oliveira et al. 2000). To date, many studies have been performed on the growth of *G. tenuistipitata var. liui* (Israel et al. 1999; Li-hong et al. 2002; Xu et al. 2009). However, information on its genetics and systematics is limited. Herein, we determined the complete mitogenome *of G. tenuistipitata* var. *liui* to provide new molecular data for genetics study.

*Gracilaria tenuistipitata* var. *liui* individual (specimen number: 2016110001) was collected from Shantou, Guangdong Province in the eastern China (23°24′19′′N, 117°3′15′′E). The high-throughput sequencing method and data processing followed Liu et al. (2017). *Gracilaria salicornia* (GenBank accession number: NC_023784) was used as the seed sequence.

The complete *G. tenuistipitata* var. *liui* (MG592728) mitogenome comprised a circular DNA molecule with the length of 25,879 bp. The overall A + T content of the complete mitogenome was 72.9%. The mitogenome contained 50 genes, including 24 protein-coding, two rRNA, 23 tRNA genes, and one unidentified open reading frame (ORF). Of the 24 protein-coding genes, 23 (95.83%) terminated with the TAA stop codon, and one (4.17%) with TAG (*rps*3 gene)). All protein-coding genes in *G. tenuistipitata* var. *liui* were found to have the start codon ATG. The lengths of two rRNA genes were 2,619 bp (*rnl*) and 1,394 bp (*rns*) respectively.

Bayesian analysis based on the complete mitogenomes of the twelve Gracilariaceae species was conducted using MrBayes v. 3. 1.2 (Huelsenbeck and Ronquist 2001). *Rhodymenia pseudopalmata* (KC875852) and *Sebdenia flabellata* (KJ398164) served as the out-group. All algae were divided into two clades: *Gracilaria* and *Gracilariopsis* (Figure 1). Phylogenetic analyses showed that *Gracilaria tenuistipitata* var. *liui* firstly clustered with *Gracilaria tenuistipitata*. The complete mitogenome data provided in this work would help us to understand *Gracilaria* evolution.

**Figure 1.**
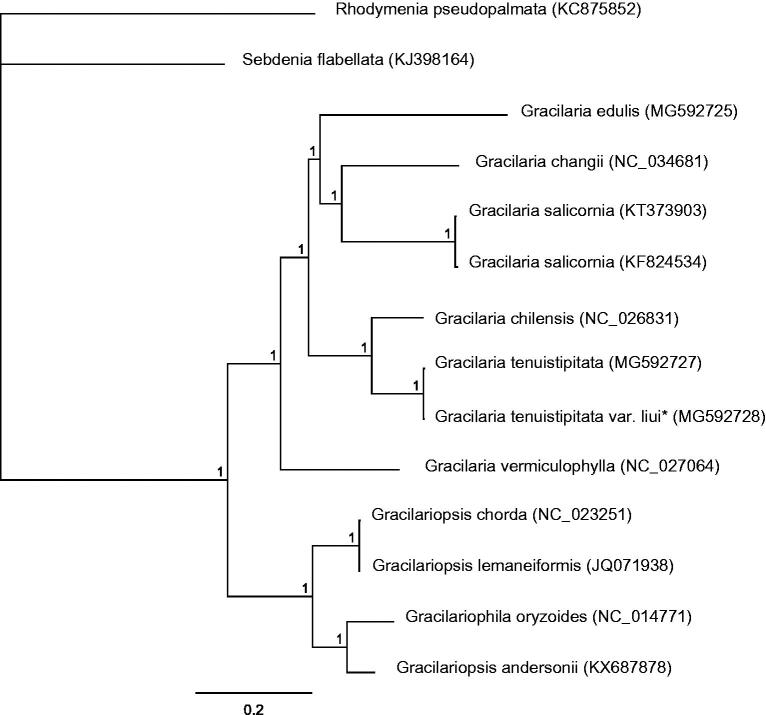
Phylogenetic tree (Bayesian inference) based on complete mitogenomes of Gracilariaceae. Support values for each node were calculated from Bayesian posterior probability (BPP). Asterisks following species names indicate newly determined mitogenomes.
